# Multimorbidity, inflammation, and disability: a longitudinal mediational analysis

**DOI:** 10.1177/2040622318806848

**Published:** 2018-10-19

**Authors:** Elliot M. Friedman, Daniel K. Mroczek, Sharon L. Christ

**Affiliations:** Department of Human Development and Family Studies, Purdue University, 1202 West State Street, West Lafayette, IN 47907, USA; Department of Psychology and Feinberg School of Medicine, Northwestern University, Evanston, IL, USA; Department of Human Development and Family Studies, Purdue University, West Lafayette, IN, USA

**Keywords:** Multimorbidity, inflammation, functional limitations, longitudinal, mediation

## Abstract

**Background:**

Using longitudinal data from the Survey of Mid-Life Development in the United States, this study examined the role of systemic inflammation in mediating the link between multimorbidity and increases in and onset of functional limitations over a 17–19 year follow-up period.

**Methods:**

Participants completed questionnaire assessments of chronic conditions and functional limitations. Interleukin-6, C-reactive protein, and fibrinogen were assayed in serum. Structural equation models were used to predict increases in and onset of functional limitations associated with baseline multimorbidity status; mediation by inflammation was also determined.

**Results:**

Multimorbidity (*versus* 0–1 conditions) predicted more functional limitations and greater odds of onset of limitations over time. Significant indirect effects showed that inflammation partially mediated the link between multimorbidity and changes in, but not onset of, limitations.

**Discussion:**

These results show that inflammation, a nonspecific marker of multiple disease conditions, explains in part the degree to which multimorbidity is disabling.

## Introduction

Despite recent declines,^1,2^ disability remains common in aging adults, resulting in substantial medical expenditures,^3^ loss of quality of life,^4,5^ and increased mortality.^6^ Beyond advancing age, the most robust predictor of disability is chronic disease, and particularly multiple chronic conditions (multimorbidity).^7,8^ Importantly, mortality related to multimorbidity appears to be largely explained by the extent to which multimorbidity is disabling,^6,9^ underscoring the need to understand the mechanisms by which multimorbidity results in functional decline and disability. The leading model for disease- and multimorbidity-related disability, the Disablement Process model,^8,10^ focuses on the specific ways in which discrete conditions may impair function. For example, heart disease may limit physical exertion because of cardiovascular dysfunction, while chronic obstructive pulmonary disease may limit functional independence because of limited breathing capacity.^8^ The current study takes a different approach, examining a biological process that many chronic conditions have in common (inflammation) as a potential mediator of the link between multimorbidity and disability.

Chronic, low-grade systemic inflammation (as opposed to large increases in inflammatory proteins that are usually indicative of acute illness or injury) has been positively linked to risk for a variety of disease conditions, and levels of inflammatory proteins rise in a linear fashion with number of chronic conditions in individuals with multimorbidity.^11^ Inflammation has also been linked to concurrent disability,^12,13^ as well as greater disability risk over time.^14,15^ Inflammation thus represents a good candidate biological mechanism linking multimorbidity and disability. The three specific inflammatory proteins examined in this study, interleukin-6 (IL-6), C-reactive protein (CRP), and fibrinogen, represent different classes of host defense factors.^16^ IL-6 is a cytokine produced by immunocompetent cells, such as monocytes and macrophages, that has diverse effects on the host response, including guiding immune cells to the site of injury or infection and instigating the acute phase response. CRP is an acute phase protein produced in the liver that directly and indirectly eliminates pathogens from the body. Fibrinogen is a clotting factor that promotes wound healing. These proteins are also biologically related, in that IL-6 is a principal driver of the synthesis and release of both CRP and fibrinogen;^17,18^ we account for their biological relatedness in the present study through the use of a latent inflammation variable for which they are indicators. We recently examined the role of these three inflammatory proteins in mediating the cross-sectional association of multimorbidity and limitations in activities of daily living (ADLs) in the nationally representative Survey of Mid-Life Development in the United States (MIDUS). We found that a latent factor for inflammation (indicated by all three inflammatory proteins) significantly mediated the relationship between multimorbidity and ADLs. Indeed, the indirect pathway from multimorbidity to disability by way of inflammation was the most robust of all indirect pathways, including those linking age, sex, race, and education to ADLs.^19^

This paper extends our previous work by examining the extent to which inflammation increases risk of later disability in aging adults with multimorbidity. Specifically, we use data from all three waves of the MIDUS study to examine the degree to which inflammation mediates the relationship between multimorbidity and both change in and likelihood of onset of ADL disability over a 17–19 year follow-up period. We predict that inflammation, again modeled as a latent factor indicated by IL-6, CRP, and fibrinogen, will partially mediate these longitudinal associations.

## Method

### Participants

Data for the current study are from all three waves of the longitudinal MIDUS study, a national survey of the physical and mental health of middle-aged and older adults. The first wave of MIDUS comprised a national probability sample of non-institutionalized English-speaking adults (*n* = 3487) living in the coterminus United States and recruited by random digit dialing. A sample of monozygotic and dizygotic twin pairs (*n* = 1914) was also recruited from a national twin registry. The first wave of MIDUS data collection (MIDUS 1) was completed in 1995–1996, and two follow-up studies (MIDUS 2 and MIDUS 3) were completed in 2004–2006 and 2013–2014, respectively. Mortality-adjusted retention was 75% from MIDUS 1 to MIDUS 2 and 77% from MIDUS 2 and MIDUS 3; a total of 1108 of the original sample of 7108 have died since study inception. All respondents completed telephone interviews and self-administered questionnaires at all three waves. The time elapsed between MIDUS 1 and MIDUS 3 participation ranged from 17 years to19 years with a mean of 18.02 years.

At the second wave (MIDUS 2), a subsample of respondents (*n* = 1054) participated in a detailed clinic-based assessment of health, disease-related biomarkers, and physiologic function (‘biomarker sample’). Participation in the biomarker sample was open to all MIDUS 2 respondents who had completed interview and questionnaire components of the study and were willing to travel to one of three regional General Clinical Research Centers (one on the West coast, one in the Midwest, and one on the East coast) for an overnight stay. Recruitment was by letter and a follow-up telephone call, and data collection was completed between 2004 and 2009. The biomarker sample was not significantly different from the main MIDUS sample on age, sex, race, marital status, or income variables, although participants were significantly more educated than the main sample.^20^ Fasting blood samples were obtained in the morning between 0800 and 1000. Serum was isolated from all samples, aliquoted, frozen at −80°C, shipped on dry ice, and stored at −80°C for assay. The analytical sample for this study consisted of the 949 biomarker sample participants who also provided data at MIDUS 3. Compared with the full biomarker sample at MIDUS, the analytical sample was younger (54.7 *versus* 58.0, *p* < 0.001), had lower levels of IL-6 (2.6 *versus* 3.7 pg/ml) and fibrinogen (337.1 *versus* 361.3 mg/dl) but not CRP, and had fewer functional limitations (1.4 *versus* 1.7, *p* < 0.001), but were otherwise comparable on key analytic variables.

Collection of data for MIDUS and analysis of those data for the current study were approved by the Institutional Review Boards at University of Wisconsin–Madison (#2014-0813) and Purdue University (#1210012882**)**. All MIDUS participants provided informed consent before being enrolled in the study, including future use of their data for analysis.

### Measures

#### Multimorbidity

Participants completed questionnaire items indicating whether or not they had experienced or received treatment for any of seven chronic medical conditions in the prior 12 months: asthma/bronchitis/emphysema, arthritis/rheumatism/joint problems, autoimmune conditions, hypertension, diabetes, neurological disorders (e.g. Parkinson’s Disease), or stroke. They also responded to two telephone interview questions asking whether that had ever been told by a doctor that they had heart problems and whether they had ever had cancer (cases of skin cancer were not included in the multimorbidity variable). Collectively, these nine conditions are among the most severe from a clinical perspective, as they are individually among the most likely to result in adverse health outcomes according to the Charlson Comorbidity Index.^21^ They are also among the most common ailments of middle-aged and older adults, and thus have appeared in multiple previous formulations of multimorbidity.^22,23^ For the main analyses, a dichotomous variable was created, indicating presence or absence of multimorbidity (0–1 conditions = 0; 2+ conditions = 1).^19,22^ Supplemental analyses were also conducted using a chronic conditions count variable with scores ranging from 0 to 9.

#### Functional limitations

Questionnaire items assessed limitations in ADLs mostly related to mobility. Respondents were asked how much health limited their ability to lift or carry groceries, climb one flight of stairs, climb several flights of stairs, bend, kneel, or stoop, walk more than a mile, walk several blocks, and walk one block. Response options ranged from 1 = Not at all to 4 = A lot. To sharpen the focus on meaningful limitations, scores for each item were dichotomized so that responses of ‘Not at all’ and ‘A little’ were scored as ‘0,’ and ‘Some’ or ‘A lot’ were scored as ‘1.’ Individual scores were then summed (range = 0–7), and the resulting total was treated as a count variable in the analyses.

#### Inflammatory proteins

Serum IL-6 from fasting blood samples was measured using high-sensitivity enzyme-linked immunosorbent assay according to manufacturer guidelines (R&D Systems, Minneapolis, MN, US) in the laboratory of Dr Christopher Coe at the University of Wisconsin–Madison. CRP and fibrinogen were measured using particle-enhanced immunonephelometric assay (BNII nephelometer, Dade Behring, Inc., Deerfield, IL, US) in the laboratory of Dr Russell Tracy at the University of Vermont. The laboratory intra- and interassay coefficients of variance for all proteins were in acceptable ranges (<10%). CRP values in excess of 10 mg/l are thought to indicate acute infectious illness, and exclusion of cases with high CRP values is recommended.^24^ We identified and excluded 30 cases with high CRP values. As is typically seen, distributions for both IL-6 and CRP were positively skewed, and data were natural log (ln)-transformed for statistical analyses.

#### Covariates

To control for potential confounds, age, sex, race, and educational attainment were all included in the models as covariates. A continuous variable was used for age (in 10-year increments for ease of interpretation), and dichotomous variables were used for sex (1 = female) and race (1= non-White). A three-level categorical variable was created for education. Respondents were asked to indicate their highest level of educational attainment using 12 categories ranging from ‘No school/some grade school’ to ‘PhD, MD, JD, or other professional degree.’ Responses were then aggregated into three categories: high-school degree or General Educational Development (GED); some college; and college degree or more, and this categorical variable was used in all analyses.

### Statistical analyses

**Figure 1.**
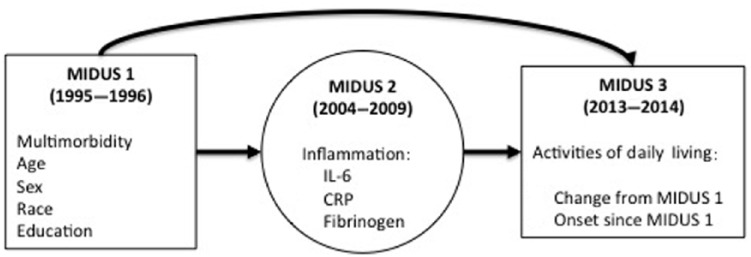
Conceptual diagram for analyses. Inflammation was modeled as a latent variable, and mediation was tested in a structural equation model. CRP, C-reactive protein; IL, interleukin; MIDUS, Survey of Mid-Life Development in the United States.

A generalized linear structural equation model (GSEM) was estimated with multimorbidity at MIDUS 1 as the predictor (along with covariates), inflammation at MIDUS 2 as a mediator, and number of ADLs at MIDUS 3 as the outcome.^25,26^ In a second analysis, ADL onset between waves 1 and 3 was modeled using a subsample of individuals who did not have any ADLs at MIDUS 1. The general path model is depicted in Figure 1. Inflammation was measured as a latent variable using ln-CRP, ln-IL6, and Fibrinogen (FGN) as indicators; the latent variable was scaled to ln-CRP.^27,28^ Paths relating the inflammation latent variable to its three indicators as well as paths linking independent variables to the inflammation mediator were estimated using linear regression. Paths from independent and mediator variables to the ADL count outcome were estimated with negative binomial regression; these paths were estimated using logistic regression for the ADL onset outcome. A likelihood ratio test indicated excess dispersion (*p* < 0.001) and therefore negative binomial regression was used to model the ADL count outcome. Coefficients from negative binomial regression models are logs of the expected counts, which can be difficult to interpret. For this reason, incident rate ratios (IRRs), a ratio of the rates of limitations in those with multimorbidity *versus* rates in those without (which can be interpreted similarly to odds ratios), are provided for effects on the ADL counts. Odds ratios from logistic models are provided for effects on ADL onset. Indirect effects were estimated using a product of moments method^29^ with bootstrap standard errors. The product of moments method estimates the indirect effect as the product of the two estimated paths in the mediation process. This method is the default in structural equation modeling and has been shown to have more statistical power than traditional, multistep tests of mediation.^30^ The product of estimates is nonlinear and the confidence intervals are asymmetric in smaller samples,^31^ therefore bootstrapping with 1000 replicates was used to obtain standard errors for the indirect effects. Maximum likelihood estimation with cluster standard errors robust to nesting of individuals within families was used.^32^

## Results

Descriptive statistics for the analytical sample are shown in Table 1.

**Table 1.** Descriptive statistics for sample (*n* = 949).

Variable	Mean (SD)	Range	Percent
Age at MIDUS 1	45.8 (11.0)	25–74	
Sex (% female)			55.5
Race (% non-White)			6.5
Educational attainment			
High school or GED			26.7
Some college			28.4
College or more			44.9
Chronic conditions (MIDUS 1)	0.6 (0.8)	0–4	
ADLs (MIDUS 1)	0.4 (1.2)	0–7	
ADLs (MIDUS 3)	1.4 (2.1)	0–7	
Inflammation (MIDUS 2)			
IL-6 (ln-transformed)	0.8 (0.7)	−1.8–2.3	
CRP (ln-transformed)	0.3 (1.1)	−2.0–2.3	
Fibrinogen (mg/dl)	341.8 (81.7)	45–789	

ADL, activity of daily living; CRP, C-reactive protein; GED, General Educational Development; IL, interleukin; ln, natural log; MIDUS, Survey of Mid-Life Development in the United States; SD, standard deviation.

The results of the primary analyses are shown in Tables 2–5. Table 2 shows the direct effects of multimorbidity and covariates on the inflammation latent factor (at MIDUS 2) and on numbers of ADL limitations at MIDUS 3 (controlling for disability at MIDUS 1). Female sex, less education, and multimorbidity were all significantly and positively related to inflammation while age and race was not. Age, female sex, multimorbidity, and inflammation were all significantly and positively related to increases in ADL limitations across the 18-year follow-up period. Specifically, compared with those with 0–1 chronic conditions, adults with multimorbidity had almost double the increase in ADL limitations (IRR = 1.93; *p* < 0.001). Each log-unit increase in inflammation predicted a 36% increase in ADL limitations. In addition, each 10-year increment in age was associated with a 28% increase in number of ADL limitations, while being female predicted 26% more of an increase than being male. Finally, each increment in the education variable (high-school graduation or GED to completion of some college, or some college to college degree or more) was associated with a 16% decrease in the rate of change in ADL limitations over time.

**Table 2.** Negative binomial regression of activity-of-daily-living limitations at the Survey of Mid-Life Development in the United States (MIDUS) 3 controlling for limitations at MIDUS 1 (*n* = 949).

	Inflammation			ADLs at MIDUS 3		
	b	95% CI		IRR	95% CI	
Age (10 years)	0.06	0.00	0.13	1.29^***^	1.17	1.41
Sex (1 = female)	0.26^***^	0.13	0.39	1.26^*^	1.03	1.55
Race (1 = non-White)	0.16	−0.11	0.42	0.87	0.58	1.30
Education	−0.15^**^	−0.23	−0.06	0.83^**^	0.73	0.94
Multimorbidity (1 = yes)	0.30^**^	0.10	0.50	1.90^***^	1.51	2.40
ADLs at MIDUS 1	0.08^**^	0.03	0.13	1.20^***^	1.14	1.27
Inflammation	–	–		1.34^***^	1.18	1.53

Beta coefficients are shown for predictors of inflammation and IRRs are shown for predictors of ADLs. Age, sex, race, education, and multimorbidity were assessed at MIDUS 1, inflammation at MIDUS 2, and ADLs at MIDUS 3.

* *p* < 0.05; ^**^p < 0.01; ^***^p < 0.001.

ADL, activity of daily living; CI, confidence interval; IRR, incidence rate ratio.

**Table 3.** Change in activities of daily living between the Survey of Mid-Life Development in the United States (MIDUS) 1 and MIDUS 3; indirect effects through inflammation (*n* = 949).

	Indirect effects		
	IRR	95% CI	
Age (10 years)	1.02	1.00	1.04
Sex (1 = female)	1.08^**^	1.02	1.14
Race (1 = non-White)	1.05	0.96	1.14
Education	0.96^*^	0.93	0.99
Multimorbidity (1 = yes)	1.09^*^	1.02	1.17
ADLs at MIDUS 1	1.02^**^	1.01	1.04

IRRs are shown.

* *p* < 0.05; ^**^p < 0.01.

ADL, activity of daily living; CI, confidence interval; IRR, incidence rate ratio.

**Table 4.** Activity-of-daily-living onset between the Survey of Mid-Life Development in the United States (MIDUS) 1 and MIDUS 3 (*n* = 786).

	Inflammation			ADL onset		
	β	95% CI		OR	95% CI	
Age (10 years)	0.10^**^	0.03	0.18	1.30^***^	1.13	1.51
Sex (1 = female)	0.24^**^	0.10	0.39	1.30	0.95	1.77
Race (1 = non-White)	0.05	−0.24	0.35	0.84	0.45	1.56
Education	−0.15^**^	−0.24	−0.06	0.77^**^	0.64	0.94
Multimorbidity (1 = yes)	0.23^$^	−0.02	0.47	3.49^***^	2.00	6.13
Inflammation	–	–		1.43^**^	1.15	1.79

Beta coefficients are shown for predictors of inflammation and ORs from logistic regression are shown for predictors of longitudinal ADL onset. Age, sex, race, education, and multimorbidity were assessed at MIDUS 1, inflammation at MIDUS 2, and ADLs at MIDUS 3.

$ *p* < 0.10; ^**^p < 0.01; ^***^p < 0.001.

ADL, activity of daily living; CI, confidence interval; OR, odds ratio.

**Table 5.** Activity-of-daily-living onset between the Survey of Mid-Life Development in the United States (MIDUS) 1 and MIDUS 3; indirect effects through inflammation (*n* = 786).

	Indirect effects		
	OR	95% CI	
Age (10 years)	1.04^$^	1.00	1.08
Sex (1 = female)	1.09^*^	1.01	1.18
Race (1 = non-White)	1.02	0.90	1.15
Education	0.95^*^	0.90	0.100
Multimorbidity (1 = yes)	1.09	0.97	1.21

$ *p* < 0.10; ^*^p < 0.05.

CI, confidence interval; OR, odds ratio.

The tests of indirect effects by way of inflammation as a mediator are shown in Table 3. With the exception of age and race, every indirect pathway showed significant mediation by inflammation, the most robust being the pathway linking multimorbidity to ADL limitations. The combined direct and indirect effect of having multimorbidity at wave one on change in ADLs between waves one and three was IRR = 2.11 (*p* < 0.001; 95% confidence interval: 1.70–2.63). Therefore, compared with respondents with 0–1 chronic conditions, individuals with multimorbidity had over twice the rate of increase in ADLs over the 18-year follow-up period, partly as a function of increased inflammation.

Table 4 is similar to Table 2, except here, the ADL outcome is the odds of incident ADL limitations between MIDUS 1 and MIDUS 3. For these analyses, the analytical sample was limited to those who had no ADL limitations at MIDUS 1 (*n* = 786). Overall, the pattern of results was similar to those for numbers of ADL limitations (see Table 2). Age, multimorbidity, and inflammation were all significantly and positively associated with greater odds of becoming disabled over time; having multimorbidity at MIDUS 1 more than tripled odds of being disabled by MIDUS 3, while greater educational attainment reduced the odds.

As shown in Table 5, inflammation significantly mediated the associations of older age (marginal significance), female sex, and less education with greater odds of becoming disabled over time. The pathway for multimorbidity was not statistically significant.

### Supplemental analyses

Two sets of analyses complement the primary models. First, we estimated linear regression models using a continuous measure of chronic conditions (values ranging from 0 to 9). The results were largely similar, although the effect sizes were substantially smaller. For example, the IRR coefficient for the indirect effect of number of chronic conditions at MIDUS 1 on ADL counts at MIDUS 3 was 1.43 (*p* < 0.01) compared with 1.93 for the dichotomous multimorbidity variable.

Second, as the multimorbidity variable consisted of nine diverse conditions that can vary in duration and severity, we conducted a sensitivity analysis to determine whether the inflammation pathway was more likely to mediate the association with ADL limitations for any single chronic condition. We repeated the GSEM analyses (predicting counts of ADL limitations) using multimorbidity variables from which a single condition had been removed each time. The results showed that the indirect effects were similar to the full multimorbidity variable when eight of the conditions were individually removed from the index. The exception was hypertension: the indirect effect for the multimorbidity variable that did not include hypertension was smaller and statistically marginally significant (*p* < 0.10).

## Discussion

As the general population continues to age, a better understanding of the diverse factors that influence the development and progression of disability will be important for insuring quality of later life. Chronic disease, and especially multimorbidity, is a leading cause of disability, although the mechanisms underlying the general relationship between multimorbidity and disability are unclear. The specific aim of this study was to test the possibility that inflammation would mediate the longitudinal association of multimorbidity and both onset and progression of disability. The results showed that across the 17–19 year follow-up period, increases in disability associated with multimorbidity were significantly mediated by inflammation. Indeed, the indirect pathway from multimorbidity to disability by way of inflammation was the most robust of all the indirect pathways tested. In contrast, onset of disability in those with multimorbidity was not mediated by inflammation. These results suggest that in middle-aged and older adults with multimorbidity, inflammation contributes more to worsening disability in those who are already disabled than to the onset of functional impairment.

These analyses consider multimorbidity generally, irrespective of the specific combinations of conditions that are characteristic of specific individuals. This perspective is consistent with calls for research into the causes and consequences of multimorbidity,^33–35^ and is supported by a wealth of data on the adverse health impact of multimorbidity, generally.^22^ We also previously showed that increased numbers of conditions were linked to higher levels of inflammation, irrespective of the specific conditions involved,^11^ and sensitivity analyses in this study confirmed that with the exception of hypertension, no single condition had more of an effect on the longitudinal indirect relationships than any other. These results suggest generally that inflammation is common to the nine conditions in the multimorbidity index and that the strength of its influence on later disability is similar across conditions.

Muscle strength may be one mechanism by which inflammation increases functional limitations. Disability risk increases with muscle weakness,^36,37^ and greater inflammation is associated with poorer performance on tasks involving muscle strength.^12,38^ Inflammatory proteins have also been shown to have direct adverse effects on skeletal muscle, including protein breakdown and impaired muscle tissue regeneration following injury.^39^ In addition, laboratory studies have shown that inflammatory proteins reduce the production of muscle cells and accelerate the loss of muscle, suggesting that even low-grade inflammation may act directly on muscle tissue and muscle-related genes to impair function.^40^

Although the focus of this study was disability related to multimorbidity, it is worth noting that inflammation also mediated the associations of other covariates, notably age, sex, and education, with onset and progression of disability. Circulating levels of inflammatory proteins like IL-6 and CRP are positively associated with advancing age, female sex, and lower socioeconomic standing.^41,42^ The current results support the possibility that inflammation may be a common pathway by which diverse factors affect disability, and they add to a growing body of research highlighting a role for inflammation in the aging process.^43^ They also suggest the potential for both exacerbating and mitigating interactions among these diverse factors, contributing to varied disability outcomes, even in those at greater risk for functional impairment. Lastly, because some markers of inflammation, notably IL-6,^44^ disease burden, and functional limitations all increase with age, the extent to which relationships among these factors is coincidental or meaningful can be challenging to establish. However, the MIDUS sample includes middle-aged adults that are less likely to show these age-related increases. The fact that inflammation mediates the association of multimorbidity and functional limitations in middle-aged as well as older adults provides clearer evidence of a specific role for inflammation as a mediator linking multimorbidity and disability.

A number of limitations temper the results. Inflammation was only measured once, meaning that it was not possible to assess either baseline levels of inflammation or change over time. It is possible that relatively higher levels of inflammation preceded multimorbidity, although this possibility does not necessarily negate a mediating role, as higher levels of inflammation predict worse outcomes in clinical patients with existing conditions.^45^ In addition, the multimorbidity variable is based on self-report of disease rather than clinical assessment, raising the possibility of inaccurate reporting. This is mostly an issue for estimating population prevalance,^46^ however, and may not affect analyses such as these, assessing long-term associations among diverse factors; if anything, inaccurate reporting would tend to undermine the strength of these associations. The nature of the data also makes it impossible to examine the degree to which these associations differ with differences in severity and duration of specific conditions. Again, though, such differences would tend to increase noise in the data and thus undermine the ability to detect the differences observed here; meaning that the strength of these associations may be underestimated. Along similar lines, and as is the case in many longitudinal studies, mortality in the sample over time was among those who were older and more impaired. This selective attrition tends to yield a longitudinal sample that is younger and more robust, making them less nationally representative. For this reason, the associations reported here may not represent those found in the population overall, although, given the nature of sample attrition, if anything, they are likely to be underestimates of the true population values. Finally, the longitudinal MIDUS sample is more ethnically and racially homogenous than the US population, meaning that these results may not apply to some sectors of the population.

These limitations are countered by significant strengths, notably mediational analyses based on long-term temporal ordering and a large age-diverse national sample. The results build on our previous work,^19^ and provide more stringent and compelling evidence that inflammation is in the pathway from multimorbidity to disability. As disability remains a concern both for aging individuals and for public health professionals, a better understanding of the processes that lead to disability in vulnerable individuals holds the potential for improving quality of later life.
